# Can physiologic colonic [^18^F]FDG uptake in PET/CT imaging predict response to immunotherapy in metastatic melanoma?

**DOI:** 10.1007/s00259-023-06327-9

**Published:** 2023-07-15

**Authors:** Christos Sachpekidis, Christoph K. Stein-Thoeringer, Annette Kopp-Schneider, Vivienn Weru, Antonia Dimitrakopoulou-Strauss, Jessica C. Hassel

**Affiliations:** 1 Clinical Cooperation Unit Nuclear Medicine, German Cancer Research Center (DKFZ), Im Neuenheimer Feld 280, 69210 Heidelberg, Germany; 2 Laboratory of Translational, Microbiome Science, Internal Medicine I, University Clinic Tuebingen, Tuebingen, Germany; 3 Department of Biostatistics, German Cancer Research Center (DKFZ), Heidelberg, Germany; 4 Department of Dermatology and National Center for Tumor Diseases (NCT), University Hospital Heidelberg, Heidelberg, Germany

**Keywords:** Metastatic melanoma, PET/CT, [^18^F]FDG colonic uptake, SUV, MTV, TLG, Immunotherapy, Gut microbiome, Lipopolysaccharide (LPS)

## Abstract

**Aim:**

The development of biomarkers that can reliably and early predict response to immune checkpoint inhibitors (ICIs) is crucial in melanoma. In recent years, the gut microbiome has emerged as an important regulator of immunotherapy response, which may, moreover, serve as a surrogate marker and prognosticator in oncological patients under immunotherapy. Aim of the present study is to investigate if physiologic colonic [^18^F]FDG uptake in PET/CT before start of ICIs correlates with clinical outcome of metastatic melanoma patients. The relation between [^18^F]FDG uptake in lymphoid cell-rich organs and long-term patient outcome is also assessed.

**Methodology:**

One hundred nineteen stage IV melanoma patients scheduled for immunotherapy with ipilimumab, applied either as monotherapy or in combination with nivolumab, underwent baseline [^18^F]FDG PET/CT. PET/CT data analysis consisted of standardized uptake value (SUV), metabolic tumor volume (MTV), and total lesion glycolysis (TLG) calculations in the colon as well as measurements of the colon-to-liver SUV ratios (CLR_mean_, CLR_max_). Visual grading of colon uptake based on a four-point scale was also performed. Moreover, the spleen-to-liver SUV ratios (SLR_mean_, SLR_max_) and the bone marrow-to-liver SUV ratios (BLR_mean_, BLR_max_) were calculated. We also measured serum lipopolysaccharide (LPS) levels as a marker for bacterial translocation and surrogate for mucosal defense homeostasis. The results were correlated with patients’ best clinical response, progression-free survival (PFS), and overall survival (OS) as well as clinical signs of colitis.

**Results:**

Median follow-up [95%CI] from the beginning of immunotherapy was 64.6 months [61.0–68.6 months]. Best response to treatment was progressive disease (PD) for 60 patients, stable disease (SD) for 37 patients, partial response (PR) for 18 patients, and complete response (CR) for 4 patients. Kaplan–Meier curves demonstrated a trend for longer PFS and OS in patients with lower colonic SUV and CLR values; however, no statistical significance for these parameters as prognostic factors was demonstrated. On the other hand, patients showing disease control as best response to treatment (SD, PR, CR) had significantly lower colonic MTV and TLG than those showing PD. With regard to lymphoid cell-rich organs, significantly lower baseline SLR_max_ and BLR_max_ were observed in patients responding with disease control than progression to treatment. Furthermore, patients with lower SLR_max_ and BLR_max_ values had a significantly longer OS when dichotomized at their median. In multivariate analysis, PET parameters that were found to significantly adversely correlate with patient survival were colonic MTV for PFS, colonic TLG for PFS, and BLR_max_ for PFS and OS.

**Conclusions:**

Physiologic colonic [^18^F]FDG uptake in PET/CT, as assessed by means of SUV, before start of ipilimumab-based treatment does not seem to independently predict patient survival of metastatic melanoma. On the other hand, volumetric PET parameters, such as MTV and TLG, derived from the normal gut may identify patients showing disease control to immunotherapy and significantly correlate with PFS. Moreover, the investigation of glucose metabolism in the spleen and the bone marrow may offer prognostic information.

**Supplementary Information:**

The online version contains supplementary material available at 10.1007/s00259-023-06327-9.

## Introduction

Over the last decade, tremendous advances have taken place in the treatment of metastatic melanoma. In particular, the introduction in clinical practice of immune checkpoint inhibitors (ICIs) has exhibited remarkable benefit in the malignancy, leading to unprecedented response and survival rates [1]. The most widely used ICIs in metastatic melanoma are the PD-1 inhibitors nivolumab and pembrolizumab, applied both as monotherapy and in combination with the CTLA-4 inhibitor ipilimumab, which represents a landmark agent, being the first ICI approved in 2011, paving the way for the wide application of ICIs in clinical routine [2–6]. Moreover, recently, a new generation of ICIs, namely, the inhibitors of the lymphocyte-activation gene 3 (LAG-3), applied in combination with PD-1 inhibitors, has yielded very promising results and has been added in the therapeutic armamentarium for patients with metastatic or unresectable melanoma [7].

Despite these indisputable improvements, the advent of novel cancer immunotherapies is also linked with some challenges. These include the heterogeneous therapeutic efficacy of these agents, which is linked with a high incidence of resistance [8], the emergence of novel imaging response patterns previously not or seldom seen with cytotoxic approaches, challenging the conventional ways of assessing treatment efficacy [9, 10], and the development of immune-related adverse events (irAEs) due to the reactivation and unleashing of T lymphocyte-mediated immune responses against tumors [11]. Therefore, the need for reliable biomarkers of efficacy and toxicity in the context of immunotherapy is crucial, since these could facilitate the tailoring of patient management with significant therapeutic and prognostic implications.

The gut microbiome has emerged as an important regulator of immunotherapy response, playing a crucial role in the interplay of tumor and immune system [12]. Several studies have highlighted the association between high gut microbiome diversity and beneficial clinical response to ICIs in various tumors [13–17]. Moreover, a linkage between baseline gut microbiome composition and ICI-associated colitis has been suggested [17, 18]. Of note, in a mouse melanoma model, anti-PD-1/CTLA-4 therapy induced translocation of bacteria into secondary lymphoid organs and tumors, which can activate dendritic cells and prime antitumor T cell responses [19]. It has also been hypothesized that increased gut permeability after ICI-induced inflammation leads to translocation of bacteria or their toxins from the gut epithelium into the circulation, which further modulates antitumor efficacies and/or ICI-induced toxicity [20]. However, associations between bacterial translocation, colonic inflammation, and ICI efficacy or toxicity have not been studied in melanoma patients.

^18^F-fluorodeoxyglucose ([^18^F]FDG) positron emission tomography/computed tomography (PET/CT) is the elective imaging modality in detection of metastatic disease in advanced melanoma [21, 22], with, moreover, an upgraded role in recent years as a tool for treatment response evaluation and prognosis, mainly in the immunotherapy setting [23–25]. In particular, apart from monitoring tumor/target lesions [26, 27], [^18^F]FDG PET/CT has been applied in the investigation of the metabolism and signs of immune activation in otherwise healthy tissues, showing promising results in prediction of response to ICIs [28–30]. In this context, several PET parameters have been recognized to unfavorably influence melanoma survival, including the number of tumor lesions [31], the intensity of tracer uptake in melanoma lesions reflected by standardized uptake value (SUV) [32, 33], volumetric parameters of tumor burden reflected by metabolic tumor volume (MTV), and total lesion glycolysis (TLG) [34–37], as well as changes in the metabolism of organs not infiltrated by tumor cells such as the colon, the bone marrow, and the spleen [30, 38–41]. Based on this knowledge, PET/CT may be potentially utilized as a non-invasive tool for the study of the metabolism and, in turn, the predictive role of the gut microbiome in patients undergoing immunotherapy.

Aim of the present study is to investigate the role of functional imaging using physiologic colonic [^18^F]FDG uptake in PET/CT in prediction of the clinical outcome of metastatic melanoma patients undergoing immunotherapy with the agent ipilimumab. The relation between [^18^F]FDG uptake in lymphoid cell-rich organs, namely, the spleen and the bone marrow, and long-term patient outcome is also assessed.

## Materials and methods

### Study population

One hundred nineteen patients (79 males, 40 females; mean age 56.4 years) with unresectable, stage IV melanoma, scheduled for ipilimumab-based therapy between February 2012 and October 2018 were enrolled in the study. Ipilimumab was applied either as monotherapy or as combination ICI treatment with nivolumab. As monotherapy ipilimumab was administered intravenously at a dose of 3 mg/kg every 3 weeks for a total of 4 doses. Respectively, the combination ICI therapy was administered as an induction of 4 cycles of nivolumab (1 mg/kg) and ipilimumab (3 mg/kg) every 3 weeks, followed by single-agent nivolumab administration (3 mg/kg) every 2 weeks.

All participants did not receive medication that can influence gut microbiota at least within 4 weeks before the baseline PET/CT. In particular, patients did not have a medication history that included probiotics, antibiotics, metformin, or chemotherapy [42]. Moreover, patients with concurrent inflammatory bowel disease were excluded. On the other hand, patients under systemic steroid therapy were not excluded from the analysis. Patients gave written informed consent to participate in the study and to have their medical records released. This is a retrospective analysis of a prospective study approved by the Ethical Committee of the University of Heidelberg (S-107/2012) and the Federal Agency for Radiation Protection (Bundesamt für Strahlenschutz, Z 5–22,463/2–2012-016).

### PET/CT data acquisition

[^18^F]FDG PET/CT was performed before the start of ICI treatment. All patients underwent whole body PET/CT 60 min after intravenous administration of maximum 250 MBq [^18^F]FDG. PET/CT studies were performed from the head to the feet with an image duration of 2 min per bed position for the emission scans. A dedicated PET/CT system (Biograph mCT, S128, Siemens Co., Erlangen, Germany) with an axial field of view of 21.6 cm with TruePoint and TrueV operated in a three-dimensional mode was used. A low-dose attenuation CT (120 kV, 30 mA) was used for attenuation correction of the PET data and for image fusion. All PET images were attenuation corrected and an image matrix of 400 × 400 pixels was used for iterative image reconstruction. Iterative image reconstruction was based on the ordered subset expectation maximization algorithm (OSEM) with six iterations and twelve subsets.

### PET/CT data analysis

Quantitative PET/CT data analysis of the colon was based on volumes of interest (VOIs) drawn over the entire extent of colonic regions with the visually, highest diffuse or segmental [^18^F]FDG uptake compared to rest colon activity if without focal tracer enhancement [43, 44]. Patients with clearly delineated, focal hypermetabolic lesions in the colon were not enrolled in the analysis, since these could represent cancerous tissue (colon metastases), given the very aggressive nature of advanced melanoma. In the cases of failure to clearly identify a colonic area of higher tracer concentration, VOIs were drawn over the cecum including at least five regions of interest (ROIs) in sequential PET/CT images. Based on this, average SUV (SUV_mean_), maximum SUV (SUV_max_), MTV, and TLG of the respective colonic areas were calculated. SUV was calculated in the respective VOIs as (radioactivity)/(injected dose/body weight). MTV (ml) was measured setting a margin threshold of 40% of SUV_max_. TLG (g) was calculated as the product of SUV_mean_ and MTV for the segmented regions (TLG = SUV_mean_ × MTV) [32]. Moreover, the SUV values of the physiologic liver parenchyma, if without disseminated metastatic disease, were measured after placement of a VOI on the right liver lobe. Based on these measurements, the colon-to-liver SUV ratios (CLR_mean_, CLR_max_) were calculated. Further, colon uptake was graded based on visual assessment of PET/CT images, according to a four-point scale, as proposed by Gontier et al.: (1) uptake less than the background hepatic activity, (2) uptake similar to that of the liver, (3) uptake moderately greater than that of the liver, and (4) intense and diffuse uptake, markedly higher than hepatic activity [45].

In an attempt to investigate lymphoid cell-rich organs, the SUV_mean_ and SUV_max_ of the physiologic spleen parenchyma as well the bone marrow were measured after placing a central VOI in the spleen and the lower thoracic vertebral bodies, respectively. On the basis of these measurements, the spleen-to-liver SUV ratios (SLR_mean_, SLR_max_) and the bone marrow-to-liver SUV ratios (BLR_mean_, BLR_max_) were calculated. VOIs were drawn using the pseudo-snake algorithm of the Pmod software [46].

### Clinical data

Clinical data were extracted from the patients’ medical records. Patients developing symptoms of colitis/diarrhea during immunotherapy were graded using the Common Terminology Criteria for Adverse Events (CTC-AE) 4.03 [47].

Patients’ best clinical response to immunotherapy was based on standard-of-care imaging (including follow-up brain MRI and [^18^F]FDG PET/CT studies). Response to therapy was defined by the following:

- response rate “RR” (responders = complete response [CR] + partial response [PR] vs non-responders = progressive disease [PD] + stable disease [SD]), and
- disease control rate “DCR” (disease control = CR + PR + SD vs no-disease control = PD).

Lipopolysaccharide (LPS) analysis from patient serum samples was performed using the Limulus amebocyte lysate (LAL) chromogenic endpoint assay (Hycult Biotech) according to the manufacturer’s guidelines. The LAL assay is among the most sensitive tests for detecting LPS as an endotoxin [48], validated in several clinical studies regarding the detection of endotoxemia in patients with gram-negative bacteremias [49], while it has also been investigated in other patient cohorts apart from infectious diseases [50].

### Statistical analysis

To investigate the relationship between the quantitative PET and binary clinical parameters, Wilcoxon rank sum test was used. Additionally, Spearman’s rank correlation was used to evaluate correlations between the continuous clinical and quantitative PET parameters as well as among the quantitative PET parameters. The association between the quantitative PET parameters and best response to treatment was investigated using the Jonckheere-Terpstra test, where best response to treatment was classified into the ordinal categories PD, SD, PR CR. Differences in survival, progression-free survival (PFS), and overall survival (OS) among groups were investigated using Kaplan–Meier plots and the log-rank test. Median follow-up was calculated using the reverse Kaplan–Meier. To investigate association between survival time of patients and multiple predictors simultaneously, the Cox proportional-hazards model was used. For parameters highly correlated with each other, such as SUV_mean_ and SUV_max_, MTV and TLG, SUV_mean_ and TLG, only one was included at a time in the model. No correction for multiple testing was performed as the study was exploratory. The results were considered significant for *p*-values less than 0.05 (*p* < 0.05). Statistical analysis was done in R (version 4.0.3).

## Results

### Patient cohort

Patient characteristics are summarized in Table 1. According to the American Joint Committee on Cancer (AJCC, 8th edition) stratification, 17 patients (14%) were classified M1a, 19 patients (16%) M1b, and 83 patients (70%) M1c. Sixty-two patients of the cohort received first-line systemic treatment with ipilimumab (with or without nivolumab), while the remaining 57 patients had received at least one systemic pretreatment. Thirty-eight patients had previously received radiotherapy. Baseline mean LDH was 304.4 U/l (median = 231.5 U/l). All included patients received treatment with ipilimumab applied either as monotherapy (*n* = 104 patients) or as combination ICI treatment with nivolumab (*n* = 15 patients).

**Table 1:** Patient characteristics and descriptive statistics of baseline PET parameters

Patient characteristics	Value
Median age, years	56.6 [14.3–84.6]
Gender	
Male	79 (66%)
Female	40 (34%)
Stage (AJCC classification)	
M1a	17 (14%)
M1b	19 (16%)
M1c	83 (70%)
ECOG performance status	
Score 0	94 (79%)
Score 1	21 (18%)
Score 2	4 (3%)
ICI treatment	
Ipilimumab	104 (87%)
Ipilimumab/nivolumab	15 (13%)
Prior systemic therapies	
No	62 (52%)
Yes	57 (34%)
Prior chemotherapy	51 (43%)
Prior radiation therapy	38 (32%)
Diarrhea	
Yes	29 (24%)
No	89 (75%)
n.d	1 (1%)
Diarrhea CTC-AE grade	
Grade 1	13
Grade 2	6
Grade 3	8
n.d	2
Systemic steroids	
Yes	44 (37%)
No	74 (62%)
n.d	1 (1%)
Baseline median LDH, U/l	231.5 [108.0–1859.0]
Baseline median S100, μg/l	0.09 [0.01–8.71]
Baseline median LPS, EU/ml	0.94 [0.00–25.73]
Best response to treatment	
PD	60 (50%)
SD	37 (31%)
PR	18 (15%)
CR	4 (3%)
Response rate (RR)§	22 (18%)
Disease control rate (DCR)†	59 (50%)
PET parameters	
Colonic SUV_mean_	2.8 [1.3–8.3]
Colonic SUV_max_	4.9 [1.6–13.9]
CLR_mean_	1.41 [0.71–3.96]
CLR_max_	1.72 [0.68–5.30]
Colonic MTV, ml	2.5 [0.3–13.5]
Colonic TLG, g	7.5 [0.9–68.2]
SLR_mean_	0.86 [0.49–1.50]
SLR_max_	0.90 [0.42–1.72]
BLR_mean_	0.99 [0.38–1.74]
BLR_max_	1.10 [0.50–2.28]
Visual grading of colonic [^18^F]FDG uptake	
Grade 1	32 (27%)
Grade 2	38 (32%)
Grade 3	34 (29%)
Grade 4	10 (8%)
n.d. (diffuse hepatic metastases)	5 (4%)

*AJCC*, American Joint Committee on Cancer; *ECOG*, Eastern Cooperative Oncology Group; *n.d*., not defined; *CTC-AE*, Common Terminology Criteria for Adverse Events; *LDH*, lactate dehydrogenase; *LPS*, lipopolysaccharide; *PD*, progressive disease; *SD*, stable disease; *PR*, partial response; *CR*, complete response; *RR*, response rate; *DCR*, disease control rate; *SUV*, standardized uptake value; *CLR*, colon-to-liver SUV ratio; *MTV*, metabolic tumor volume; *TLG*, total lesion glycolysis; *SLR*, spleen-to-liver SUV ratio; *BLR*, bone marrow-to-liver SUV ratio; *n.d.*, not defined

^§^ RR = CR + PR

^†^ DCR = CR + PR + SD

### PET/CT findings

Quantitative, VOI-based, tracer uptake calculations led to yielding of baseline colonic SUV_mean_, SUV_max_, MTV, and TLG values. Moreover, the respective ratios CLR_mean_, CLR_max_, SLR_mean_, SLR_max_, BLR_mean_, and BLR_max_ were calculated. The descriptive statistics of baseline PET parameters are presented in Table 1.

Moderate but significant correlations were observed between colonic and splenic [^18^F]FDG uptake both for SUV_mean_ (*r* = 0.27; *p* = 0.005) and SUV_max_ (*r* = 0.39; *p* < 0.001). Moreover, a significant correlation was observed between colonic and bone marrow uptake for SUV_max_ (*r* = 0.30; *p* = 0.002). Colonic [^18^F]FDG uptake showed a significant correlation with baseline LDH plasma levels, both for SUV_mean_ (*r* = 0.21; *p* = 0.025) and SUV_max_ (*r* = 0.25; *p* = 0.007). On the other hand, volumetric colonic PET metrics demonstrated no significant correlation with LDH, either for MTV (*p* = 0.221) or TLG (*p* = 0.289). Further, there were no significant correlations between quantitative colonic PET parameters and baseline S100.

**Fig. 1:**
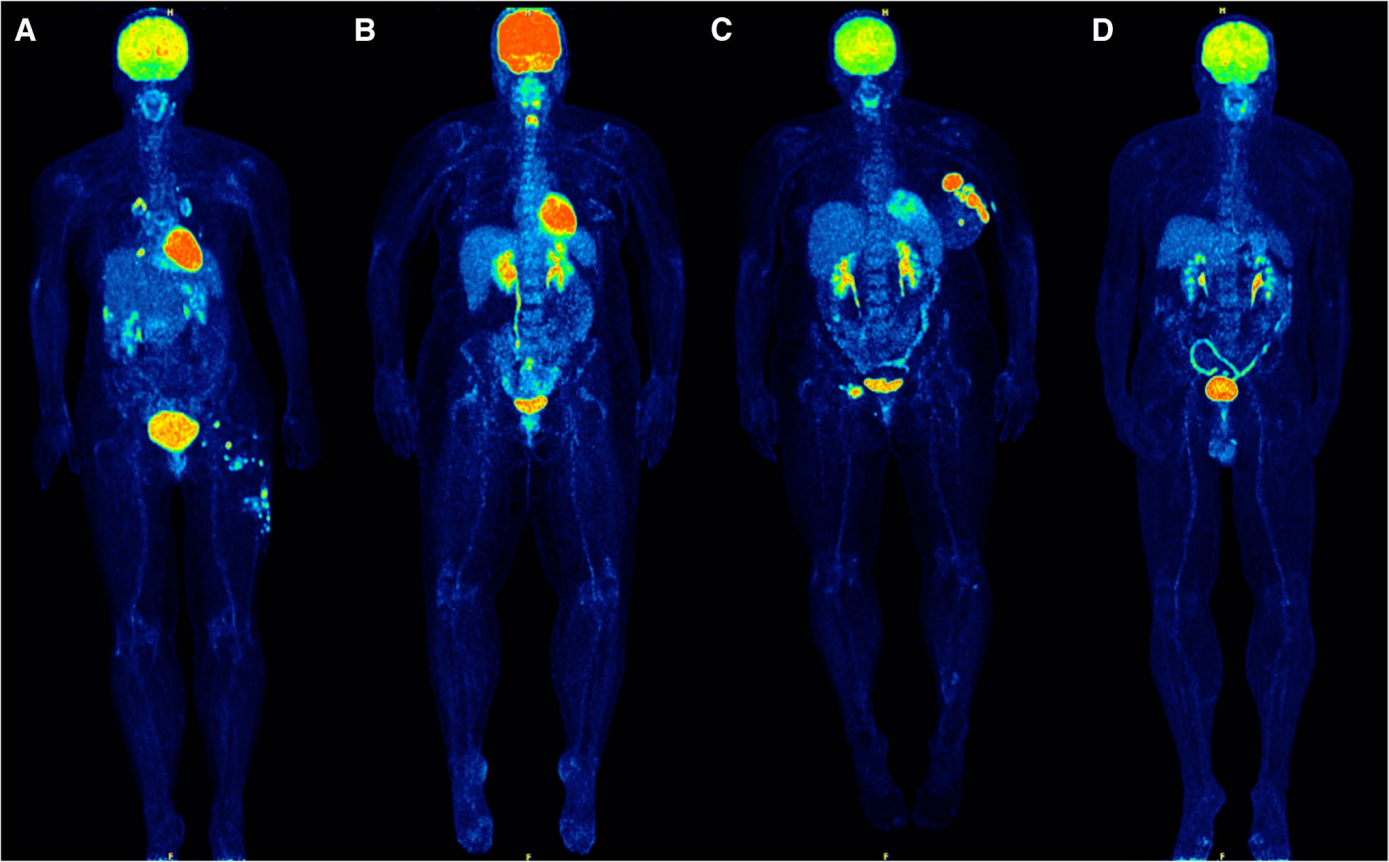
PET/CT examinations (maximal intensity projection images) of melanoma patients before initiation of ipilimumab-based immunotherapy, exhibiting different levels of colonic [.^18^F]FDG uptake. Based on visual assessment, colon uptake can be graded according to a four-point scale, as proposed by Gontier et al. [45]: 1. uptake less than the background hepatic activity (**A**), 2. uptake similar to that of the liver (**B**), 3. uptake moderately greater than that of the liver (**C**), 4. intense and diffuse uptake, markedly higher than hepatic activity (**D**)

With regard to visual grading of [^18^F]FDG uptake, this was performed in 114/119 patients, since in five patients (4%) the scaling was not feasible due to diffuse hepatic metastases. The results of the application of the four-point tracer scaling system are presented in Table 2. Figure 1 shows examples of patients with different levels of colonic [^18^F]FDG uptake before treatment.

**Table 2:** Multivariate Cox regression analysis of clinical parameters and PET metrics derived from the colon and the spleen. Since the PET parameters colonic MTV and TLG are highly correlated, two models were applied, with only one of them being included at a time in the model (model 1 including MTV and model 2 including TLG)

	Model 1 (incl. colonic MTV)		Model 2 (incl. colonic TLG)	
Parameters	HR (95% CI)	*p*-value	HR (95% CI)	*p*-value
*Progression-free survival*				
AJCC				
High AJCC	1.542 (0.884–2.690)	0.13	1.534 (0.874–2.690)	0.14
ECOG score				
High ECOG score	1.334 (0.663–2.684)	0.42	1.254 (0.626–2.511)	0.52
LDH	1.002 (1.000–1.003)	< 0.01*	1.001 (1.000–1.003)	< 0.01*
LPS	0.901 (0.827–0.982)	0.02*	0.905 (0.830–0.986)	0.02*
Colonic MTV	1.087 (1.007–1.175)	0.03*	1.025 (1.001–1.050)	0.04*
SLR_max_	1.953 (0.383–9.953)	0.42	2.069 (0.403–10.619)	0.38
*Overall survival*				
AJCC				
High AJCC	2.775 (1.357–5.676)	< 0.01*	2.764 (1.346–5.674)	< 0.01*
ECOG score				
High ECOG score	2.992 (1.464–6.117)	< 0.01*	3.004 (1.468–6.144)	< 0.01*
LDH	1.002 (1.001–1.003)	< 0.01*	1.002 (1.001–1.003)	< 0.01*
LPS	0.889 (0.794–0.996)	0.04*	0.888 (0.793–0.994)	0.04*
Colonic MTV	0.987 (0.904–1.077)	0.77	0.999 (0.973–1.026)	0.95
SLR_max_	0.999 (0.160–6.248)	0.99	0.993 (0.160–6.167)	0.99

^*^ Statistically significant correlation

*HR*, hazard ratio; *95% CI*, 95% confidence intervals; *MTV*, metabolic tumor volume; *TLG*, total lesion glycolysis; *LDH*, lactate dehydrogenase; *LPS*, lipopolysaccharide; *AJCC*, American Joint Committee on Cancer; *ECOG*, Eastern Cooperative Oncology Group; *SLR*_*max*_, spleen-to-liver SUV_max_ ratio

High AJCC is defined by M1c patients (vs. M1a and M1b)

High ECOG score is defined by score 1 and score 2 (vs. score 0)

Since the administration of steroids may influence [^18^F]FDG uptake in the gut, we subcategorized the patients based on steroid usage at the time of PET/CT in order to assess its potential effect on colonic uptake. No significant difference was observed between the two groups for any PET parameter studied, in terms of both quantitative and visual analysis.

### Diarrhea and colitis

A total of 29 patients (24%) suffered from clinically significant diarrhea during immunotherapy: thirteen patients had grade-1, six patients grade-2, and eight patients grade-3 diarrhea. For two patients the grade of diarrhea was not defined (Table 1). None of the quantitative PET parameters, namely, colonic SUV_mean_ (*p* = 0.866), SUV_max_ (*p* = 0.712), CLR_mean_ (*p* = 0.567), CLR_max_ (*p* = 0.248), colonic MTV (*p* = 0.414), or TLG (*p* = 0.448) showed any significant difference between patients suffering from clinical diarrhea and those who did not. The same applies also for the visual grading of colonic [^18^F]FDG uptake.

Further, the patients with clinically significant diarrhea during ICIs treatment were dichotomized based on the grade of the symptoms (grade 1/grade 2 vs. grade 3). No significant differences were found between the two patient groups regarding PET parameters, namely, colonic SUV_mean_ (*p* = 0.658), SUV_max_ (*p* = 0.360), CLR_mean_ (*p* = 0.531), CLR_max_ (*p* = 0.334), colonic MTV (*p* = 0.488), and TLG (*p* = 0.334).

### LPS levels in the peripheral blood

The mean LPS levels in patient sera before start of treatment were 1.99 (EU/ml) (median = 0.94 EU/ml). There were no significant differences in LPS levels between patients suffering from diarrhea and those who did not (*p* = 0.137). Similarly, when patients were dichotomized based on the grade of diarrhea (grade 1/grade 2 vs. grade 3), no differences were observed in LPS (*p* = 0.759). In addition, no significant correlation was observed between LPS levels and the quantitative PET parameters colonic SUV (SUV_mean_, *p* = 0.828; SUV_max_, *p* = 0.505), CLR values (CLR_mean_, *p* = 0.146; CLR_max_, *p* = 0.454), colonic MTV (*p* = 0.221), or TLG (*p* = 0.289).

### Best response to treatment

Among the 119 patients, best response to treatment was PD for 60 patients, SD for 37 patients, PR for 18 patients, and CR for four patients. None of the quantitative colonic PET parameters SUV_mean_ (*p* = 0.608), SUV_max_ (*p* = 0.388), CLR_mean_ (*p* = 0.98), CLR_max_ (*p* = 0.271), MTV (*p* = 0.091), or TLG (*p* = 0.139) demonstrated significant association with best response, when this was divided in four groups (PD, SD, PR, CR). Moreover, no association was demonstrated between response to treatment and visual scaling of [^18^F]FDG uptake in the gut in four grades (*p* = 0.723).

When response to therapy was defined by “RR,” no differences between responders (PR, CR) and non-responders (PD, SD) were demonstrated in the parameters colonic SUV_mean_ (*p* = 0.406), colonic SUV_max_ (*p* = 0.329), CLR_mean_ (*p* = 0.528), CLR_max_ (*p* = 0.774), colonic MTV (*p* = 0.642), or colonic TLG (*p* = 0.901).

On the other hand, according to the “DCR”-based dichotomization, patients showing disease control (SD, PR, CR) had lower colonic MTV (*p* = 0.051) and TLG (*p* = 0.049) than those showing no-disease control (PD). In contrary, no differences were demonstrated between the two patient groups for the parameters colonic SUV_mean_ (*p* = 0.379), colonic SUV_max_ (*p* = 0.146), CLR_mean_ (*p* = 0.853), and CLR_max_ (*p* = 0.14).

Regarding the PET data derived from the spleen and the bone marrow, significantly lower SLR_max_ (*p* = 0.026) and BLR_max_ (*p* = 0.03) values were observed in patients responding with disease control to treatment compared to those with PD. No other significant differences or correlations were observed for these parameters with regard to best response to treatment.

We also investigated the association between baseline LPS levels and patient response, without, however, revealing any significant correlation according to either the RR-based (*p* = 0.955) or DCR-based (*p* = 0.795) approach.

### Patient survival

Median follow-up [95%CI] of the patient cohort from the beginning of immunotherapy was 64.6 months [61.0–68.6 months]. Median PFS of the whole cohort was 3.7 months [3.0–5.0 months], while median OS was 17.3 months [12.6–30.3 months].

With regard to baseline clinical factors, Kaplan–Meier analysis revealed an adverse, non-significant trend for PFS (*p* = 0.07) and a significant effect on OS (*p* < 0.01) for patients in higher M stage (AJCC classification). Further, patients with higher Eastern Cooperative Oncology Group (ECOG) performance status score demonstrated a non-significant trend for shorter PFS (*p* = 0.09) and a marginally shorter OS (*p* = 0.05). Previous administration of radiotherapy was associated with a significantly shorter OS (*p* = 0.01), while LDH plasma levels had a marginally significant adverse effect on PFS (*p* = 0.05) and a significant effect on OS (*p* < 0.001). All rest clinical parameters assessed (S-100, previous administration of systemic therapies, LPS levels) had no significant effect on survival.

**Fig. 2:**
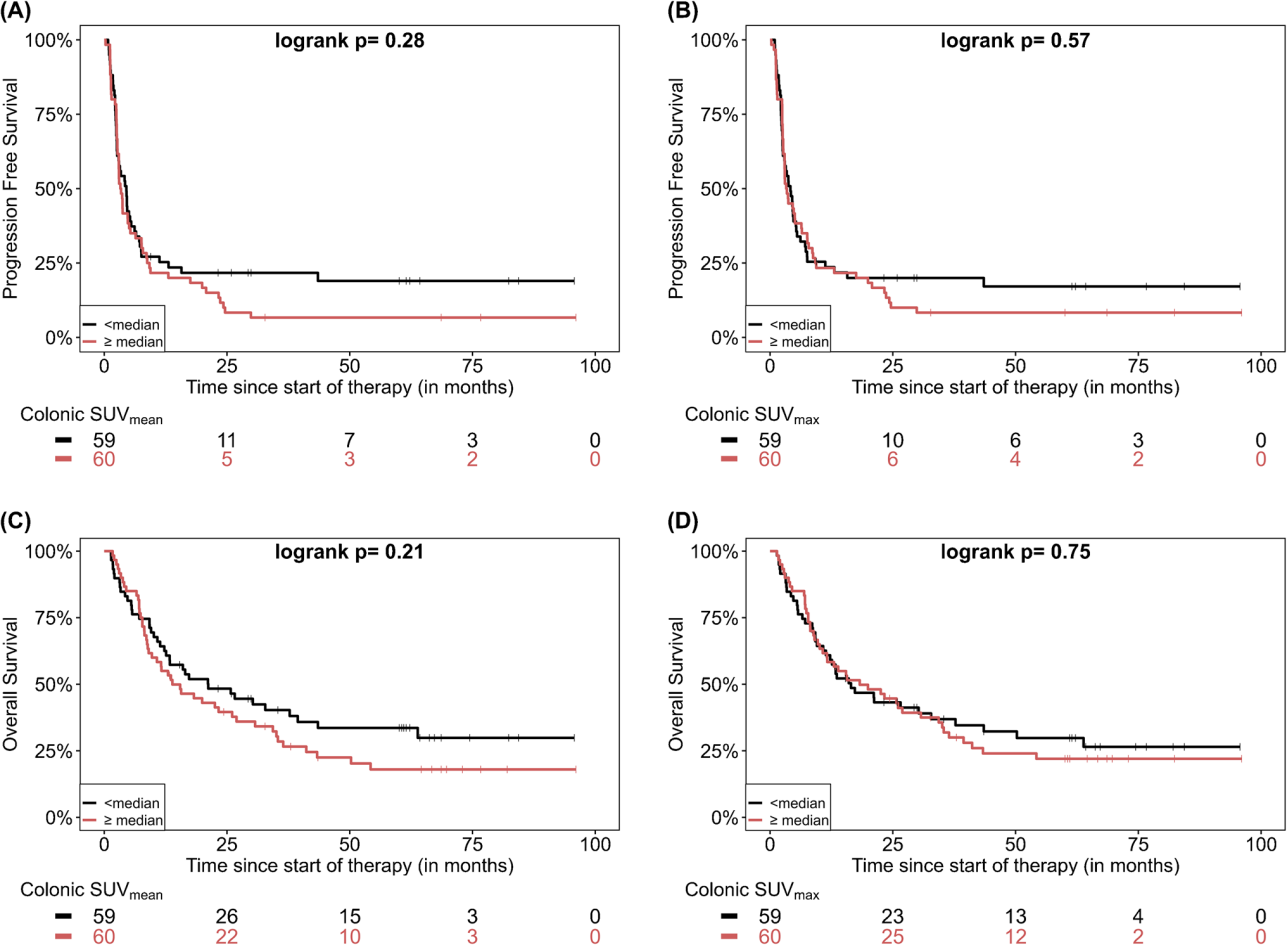
Kaplan–Meier estimates of PFS according to colonic SUV_mean_ (**A**) and SUV_max_ (**B**) as well as estimates of OS according to colonic SUV_mean_ (**C**) and SUV_max_ (**D**). The numbers of patients at risk in each group and for the respective time points are shown below the plots

**Fig. 3:**
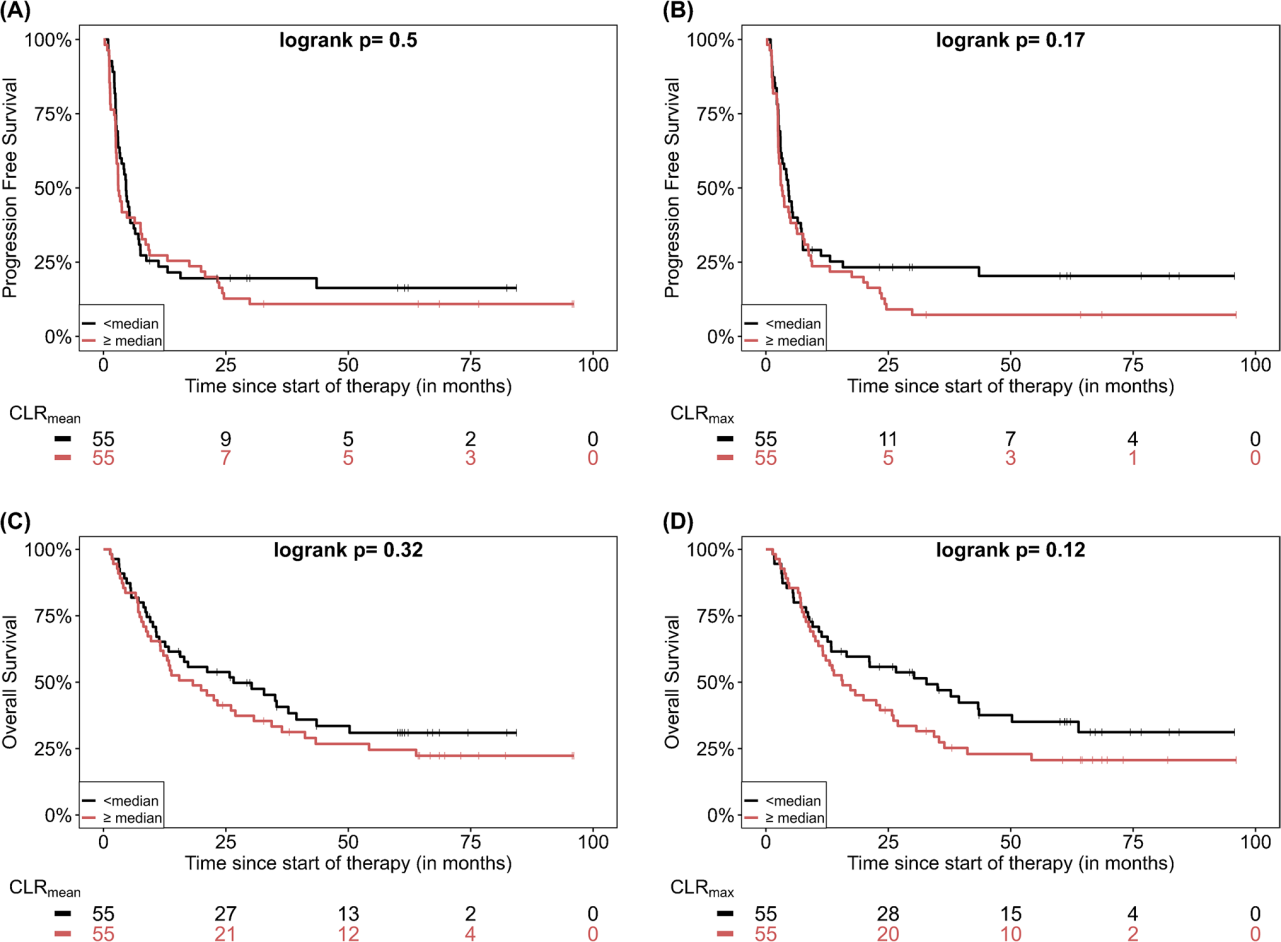
Kaplan–Meier estimates of PFS according to CLR_mean_ (**A**) and CLR_max_ (**B**) as well as estimates of OS according to CLR_mean_ (**C**) and CLR_max_ (**D**). The numbers of patients at risk in each group and for the respective time points are shown below the plots

**Fig. 4:**
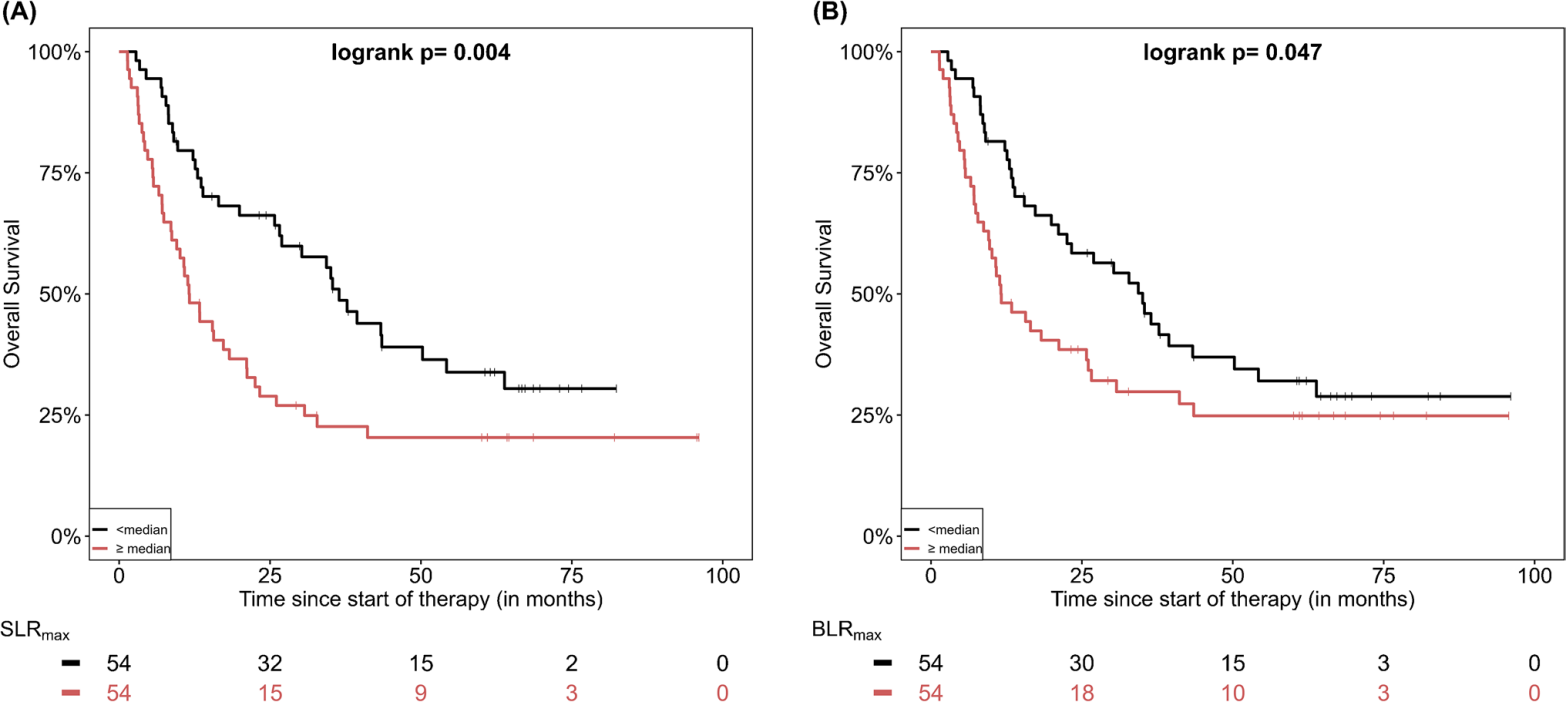
Kaplan–Meier estimates of OS according to SLR_max_ (**A**) and BLR_max_ (**B**). The numbers of patients at risk in each group and for the respective time points are shown below the plots

The effect of quantitative PET parameters on patient survival was also studied by means of Kaplan–Meier analysis. However, no statistically significant differences in survival were demonstrated for any of the studied parameters. The detailed results of survival analysis based on PET quantitative data derived from colonic tissue are presented in Supplementary Table 1. The respective curves demonstrated a trend for longer PFS and OS in patients with lower colonic SUV and CLR; however, the log-rank test revealed no statistical significance (Figs. 2 and 3). Also the four-grade visual scaling of colonic [^18^F]FDG uptake had no significant effect either on PFS (*p* = 0.88) or OS (*p* = 0.17). Further, no optimal cut-offs for these parameters regarding PFS or OS prediction could be identified using maximally selected rank statistics. With regard to the quantitative parameters derived from the spleen and the bone marrow, a significantly longer OS was observed for patients with lower than median SLR_max_ (*p* = 0.004) and BLR_max_ (*p* = 0.047) (Supplementary Table 2; Fig. 4).

A further Kaplan–Meier sub-analysis was performed on patients’ survival data based on the combination of splenic and colonic uptake, as well as bone marrow and colonic uptake. The respective curves demonstrated a trend for shorter PFS and OS in patients with higher uptake in both organs, with the log-rank test partly revealing statistical significance (for the combination of splenic and colonic SUV_max_) or borderline significance (for the combination of splenic and colonic SUV_mean_) (Supplementary Figs. 1 and 2).

In multivariable analysis, effect on survival of quantitative PET and clinical parameters was assessed simultaneously. In terms of PFS, the analysis revealed that high values of the parameters LDH, colonic MTV, colonic TLG, and BLR_max_ significantly adversely correlated with survival, while high LPS levels significantly favorably influenced PFS. With regard to OS, high M stage, ECOG status, LDH, and BLR_max_ correlated significantly adversely with patient survival, while high LPS levels significantly favorably influenced OS (Tables 2 and 3).

**Table 3:** Multivariate Cox regression analysis of clinical parameters and PET metrics derived from the colon and the bone marrow. Since the PET parameters colonic MTV and TLG are highly correlated, two models were applied, with only one of them being included at a time in the model (model 1 including MTV and model 2 including TLG)

	Model 1 (incl. colonic MTV)		Model 2 (incl. colonic TLG)	
Parameters	HR (95% CI)	*p*-value	HR (95% CI)	*p*-value
*Progression-free survival*				
AJCC				
High AJCC	1.664 (0.920–3.009)	0.09	1.616 (0.891–2.930)	0.11
ECOG score				
High ECOG score	1.446 (0.731–2.859)	0.29	1.345 (0.681–2.654)	0.39
LDH	1.004 (1.002–1.006)	< 0.01*	1.003 (1.001–1.006)	< 0.01*
LPS	0.895 (0.818–0.979)	0.02*	0.904 (0.827–0.989)	0.03*
Colonic MTV	1.129 (1.040–1.225)	< 0.01*	1.029 (1.005–1.053)	0.02*
BLR_max_	2.144 (1.020–4.505)	0.04*	1.805 (0.878–3.710)	0.11
*Overall survival*				
AJCC				
High AJCC	3.165 (1.481–6.767)	< 0.01*	3.166 (1.472–6.813)	< 0.01*
ECOG score				
High ECOG score	3.121 (1.520–6.406)	< 0.01*	3.120 (1.520–6.404)	< 0.01*
LDH	1.004 (1.001–1.006)	< 0.01*	1.004 (1.001–1.006)	< 0.01*
LPS	0.890 (0.796–0.995)	0.04*	0.890 (0.797–0.994)	0.04*
Colonic MTV	1.001 (0.917–1.093)	0.98	1.000 (0.974–1.026)	0.99
BLR_max_	2.605 (1.035–6.555)	0.04*	2.604 (1.031–6.578)	0.04*

^*^ Statistically significant correlation

*HR*, hazard ratio; *95% CI*, 95% confidence intervals; *MTV*, metabolic tumor volume; *TLG*, total lesion glycolysis; *LDH*, lactate dehydrogenase; *LPS*, lipopolysaccharide; *AJCC*, American Joint Committee on Cancer; *ECOG*, Eastern Cooperative Oncology Group; *BLR*_*max*_, bone marrow-to-liver SUV_max_ ratio

High AJCC is defined by M1c patients (vs. M1a and M1b)

High ECOG score is defined by score 1 and score 2 (vs. score 0)

## Discussion

With the current study we investigated the potential contribution of the intensity of physiologic pretreatment colonic [^18^F]FDG PET uptake in prediction of the clinical outcome of metastatic melanoma patients undergoing immunotherapy with the agent ipilimumab. In addition, the relation between [^18^F]FDG uptake in non-tumoral immune and hematopoietic organs and long-term patient outcome was assessed. To our knowledge, this is the largest study to date investigating these relationships. The major findings from our analysis are the following: Firstly, no statistically significant correlation was observed between baseline colonic [^18^F]FDG uptake and clinical signs of colitis during immunotherapy or LPS levels in the peripheral blood as a marker for gut bacteria translocation and surrogate for gut mucosal integrity*.* Secondly, no significant correlation was observed between colonic [^18^F]FDG uptake, as assessed by SUV values, and patient survival, although the respective Kaplan–Meier curves demonstrated a trend for longer PFS and OS in patients with lower uptake values. Thirdly, the volumetric colonic PET parameters (MTV, TLG) were lower in patients showing disease control to immunotherapy compared to those with PD. Moreover, a significantly longer OS was observed in patients with lower baseline SLR_max_ and BLR_max_ values when dichotomized at their median. And finally, in multivariate analysis, colonic MTV, colonic TLG, and BLR_max_ significantly adversely correlated with patient survival.

[^18^F]FDG uptake in the colon shows a wide variety both in distribution and intensity. In particular, a high colonic tracer uptake is quite frequent on PET imaging and often relates to a physiologic origin. Several hypotheses have been suggested to explain this finding, such as [^18^F]FDG uptake by the smooth muscle and the superficial mucosal cells in the intestinal wall or the lymphoid tissue of the gut as well as intraluminal tracer excretion; however, there still exists a lack of understanding regarding its exact underlying mechanism [45, 51]. Moreover, although colonic [^18^F]FDG uptake may be correlated with different concentrations of specific intestinal bacteria [21, 42, 52–54], it is unclear to what extent the PET/CT method can reliably reveal the complexity of the microbiome composition.

We showed that baseline [^18^F]FDG gut uptake, evaluated by different approaches, both quantitative, such as colonic SUV (either as an absolute value or in relation to liver uptake), and qualitative, based on visual evaluation of PET images [45], neither correlated with best clinical response to treatment nor predicted patient survival at a significant level. This can be attributed to the non-specific and normally, highly variable uptake of [^18^F]FDG in the organ. Notably, however, the respective Kaplan–Meier curves of SUV and CLR revealed a non-significant trend for longer PFS and OS in patients with lower colonic [^18^F]FDG uptake.

Interestingly, the metabolic volumetric parameters MTV and TLG from the normal gut were lower in patients showing disease control compared to those with disease progression. This finding is considerable, since in immunotherapy disease control represents a satisfactory outcome, given that SD can be durable, with survival rates comparable to those associated with response [55–57]. This contrasts the traditional approaches for definition of treatment efficacy applied in conventional chemotherapy. In line with the previous studies, multivariate analysis revealed that colonic MTV and TLG significantly adversely affected patients’ PFS, suggesting the role of these parameters as potential prognosticators of immunotherapy outcome.

In a smaller study of 14 patients, Boursi et al. also investigated the role of physiologic colonic [^18^F]FDG uptake as a possible predictor for response to ipilimumab in melanoma. They reported that total colonic SUV_max_ of individuals showing CR to ipilimumab was significantly lower than those without CR (PR or PD), concluding that physiologic colonic [^18^F]FDG uptake may predict CR to immunotherapy [29]. Apart from the cohort size, which was markedly smaller than in our analysis, and the follow-up period, which was not provided by the authors, a considerable difference between the above-mentioned study and ours lies in the PET/CT image analysis. Boursi et al. calculated the total colonic SUV after manually drawing ROIs around the outer boundaries of the four colonic regions (cecum and ascending, transverse, descending, and rectosigmoid) and, subsequently, combining the regional measurements. On the other hand, our analysis focused on the assessment of [^18^F]FDG uptake, including volumetric measurements, in colonic regions with the highest diffuse or segmental [^18^F]FDG uptake compared to rest colon activity, or alternatively in the cecum, in the case of failure to visually identify a colonic region with distinctly increased tracer concentration. The choice of the cecum was based on previous [^18^F]FDG PET/CT studies, which highlighted this colonic region as a site potentially exhibiting signs of lymphocyte activation, and showing, moreover, a correlation with patient survival during immunotherapy and microbiome diversity [30, 58]. Although it cannot be ascertained which approach performs better, we preferred to evaluate intestinal areas of increased tracer concentration instead the whole colon in order to assess the gut areas with the highest metabolic activity. Moreover, the herein applied approach seems more operator friendly and less time intensive to implement in daily clinical practice.

Another study in the field, albeit at a different tumor and with different therapeutic agents, was recently published by Cvetkovic et al. The authors studied a group of patients with advanced NSCLC treated with anti-PD-1/PD-L1 monotherapy or in combination with chemotherapy, and found that a lower colon [^18^F]FDG uptake on PET/CT at baseline was associated with better clinical outcomes [58]. Also in this study, the method of analysis of the PET/CT images was more complex than ours. In particular, colon segmentation was based on division of the organ in five anatomic segments (cecum, right, transverse, left, and rectosigmoid), followed by manual contouring of each portion separately on axial CT images, which were then transposed onto the corresponding axial PET images.

Apart from the assessment of colonic metabolism, our analysis also involved quantitative calculations of [^18^F]FDG uptake in lymphoid cell-rich organs, namely, the spleen and the bone marrow. The main finding here was the significantly shorter OS for patients with high SLR_max_ and BLR_max_, when dichotomized at their median, while in multivariate analysis, the parameter BLR_max_ was found to significantly adversely correlate both for PFS and OS. Moreover, the Kaplan–Meier analysis based on the combination of tracer uptake in the colon and lymphoid cell-rich organs demonstrated a trend for shorter PFS and OS in patients with higher uptake, with the log-rank test partly revealing statistical significance or borderline significance. The finding that hematopoietic and lymphoid tissue metabolism, investigated by means of PET/CT, correlates with unfavorable clinical outcomes is in line with recent works in the field [35, 59, 60], suggesting their predictive role in ICI treatment. At the same time, however, a less contributory role of the baseline metabolism of these organs in prediction of response to immunotherapy has also been demonstrated [39]. In this respect, although our results highlight the potential role of these imaging metrics as predictive indicators, they should be cautiously interpreted, since there exists a clear need for prospective and translational studies correlating glucose metabolism in these organs with the pathophysiology of immune activation elicited by ICIs [61]. Overall, however, the extraction and investigation of PET biomarkers related to the host immune system is gradually gaining importance as a potential surrogate marker of therapeutic response.

Our study has some limitations. First, this is a single-center retrospective analysis of prospectively acquired data. This resulted in the lack of certain evidence, such as gut microbiome data, stool samples, as well as other clinical and laboratory data, that would allow a further elucidation of the association between [^18^F]FDG uptake and microbiome composition. Thus, a validation of these findings in larger patient cohorts, ideally studied in the context of a multicenter, prospective trial, would be required. Second, our analysis was focused on ipilimumab-based immunotherapy, since colitis has been mainly associated with anti-CTLA-4 agents, occurring in 10–20% of patients undergoing this type of treatment [62]. Although, since the introduction of anti–PD-1 antibodies, ipilimumab has been less commonly used as first-line monotherapy, the agent is still used in combination with nivolumab as first-line therapy in patients with advanced melanoma and as subsequent therapy in patients with disease progression after single-agent anti-PD-1 treatment [63]. Finally, no exact volumetric PET calculations of whole-body tumor burden were performed in the context of the present analysis. Our group is, however, in the process of developing and evaluating a respective tool for whole-body metabolic tumor calculations, which will be the topic of a future work [64].

## Conclusion

In an attempt to investigate the role of baseline, physiologic colonic [^18^F]FDG uptake in prediction of immunotherapy outcome, we studied by means of PET/CT a cohort of 119 stage IV melanoma patients undergoing treatment with the agent ipilimumab. At a median follow-up of 64.6 months [61.0–68.6 months] after treatment, start physiologic colonic [^18^F]FDG uptake, as assessed by SUV, did not independently predict patient survival, although a non-significant trend for longer PFS and OS was observed in patients with lower colonic [^18^F]FDG uptake. Moreover, colonic [^18^F]FDG uptake was not correlated with either clinical signs of colitis during immunotherapy or LPS levels in the peripheral blood. On the other hand, volumetric PET parameters, such as MTV and TLG, derived from the normal gut significantly correlated with disease control to immunotherapy, suggesting their potential role as prognosticators of response to ICIs. Further, the analysis of the spleen and bone marrow metabolism showed considerable promise for the long-term prediction of treatment outcome through the demonstration of a significant correlation between SLR_max_ and BLR_max_ values (when dichotomized at their median) with OS. Notably, in multivariate analysis, the PET parameters colonic MTV, colonic TLG, and BLR_max_ were found to significantly adversely correlate with patient survival.

### Supplementary Information

Below is the link to the electronic supplementary material.

Supplementary file1 (DOCX 2266 KB)

## Data Availability

The datasets generated during and/or analyzed during the current study are available from the corresponding author on reasonable request.
