# Supplementary material for: Can physiologic colonic [18F]FDG uptake in PET/CT imaging predict response to immunotherapy in metastatic melanoma?
Source: Eur J Nucl Med Mol Imaging. 2023 Jul 15;50(12):3709–22. doi: 10.1007/s00259-023-06327-9 (PMC10547632; doi:10.1007/s00259-023-06327-9)
Supplement: Supplementary file 1 — Supplementary file1 (DOCX 2266 KB) [file 259_2023_6327_MOESM1_ESM.docx]

**Supplementary Table 1** Results of survival (Kaplan-Meier) analysis based on quantitative data derived from physiologic non-tumoral colonic tissue.

| **Parameter** |  | **Baseline PET/CT** | | | |
| --- | --- | --- | --- | --- | --- |
|  |  | **Median PFS [95% CI]** | ***p value*** | **Median OS [95% CI]** | ***p value*** |
| **colonic SUV_mean_** | <median | 4.5 months [2.6 – 6.5] | 0.28 | 21.2 months [12.6 – 43.5] | 0.21 |
|  | ≥median | 3.4 months [3.0 – 5.3] |  | 13.9 months [9.7 – 30.7] |  |
| **colonic SUV_max_** | <median | 4.1 months [2.6 – 5.5] | 0.57 | 16.5 months [11.4 – 43.5] | 0.75 |
|  | ≥median | 3.4 months [3.0 – 6.5] |  | 18.3 months [11.6 – 35.1] |  |
| **CLR_mean_** | <median | 4.6 months [3.4 – 6.5] | 0.5 | 26.6 months [13.3 – 50.3] | 0.32 |
|  | ≥median | 3.0 months [2.7 – 7.6] |  | 18.3 months [12.2 – 34.3] |  |
| **CLR_max_** | <median | 4.6 months [3.2 – 7.3] | 0.17 | 32.8 months [13.4 – 63.8] | 0.12 |
|  | ≥median | 3.3 months [2.7 – 6.4] |  | 15.7 months [11.6 – 26.9] |  |
| **colonic MTV** | <median | 3.7 months [3.0 – 7.1] | 0.44 | 16.0 months [10.8 – 36.4] | 0.97 |
|  | ≥median | 3.4 months [2.6 – 5.5] |  | 17.3 months [11.4 – 34.3] |  |
|  | <median | 3.6 months [2.9 – 5.3] |  | 15.7 months [12.6 – 35.1] |  |
| **colonic TLG** |  |  | 0.78 |  | 0.89 |
|  | ≥median | 3.7 months [2.7 – 6.4] |  | 19.9 months [10.1 – 35.4] |  |

SUV, standardized uptake value; CLR, colon-to-liver SUV ratio; MTV, metabolic tumor volume; TLG, total lesion glycolysis; PFS, progression-free survival; OS, overall survival.

**Supplementary Table 2** Results of survival (Kaplan-Meier) analysis based on quantitative data derived from non-tumoral immune organs.

| **Parameter** |  | **Baseline PET/CT** | | | |
| --- | --- | --- | --- | --- | --- |
|  |  | **Median PFS [95% CI]** | ***p value*** | **Median OS [95% CI]** | ***p value*** |
| **SLR_mean_** | <median | 3.7 months [3.0 – 7.6] | 0.85 | 25.8 months [15.5 – 41.2] | 0.46 |
|  | ≥median | 4.2 months [2.9 – 6.4] |  | 17.3 months [11.6 – 36.4] |  |
| **SLR_max_** | <median | 4.9 months [3.7 – 8.8] | 0.12 | 36.4 months [26.9 – 63.8] | 0.004* |
|  | ≥median | 2.9 months [2.5 – 5.3] |  | 11.6 months [8.7 – 21.2] |  |
| **BLR_mean_** | <median | 3.7 months [2.7 – 7.1] | 0.21 | 19.1 months [13.0 – 35.4] | 0.47 |
|  | ≥median | 4.4 months [3.0 – 13.1] |  | 26.6 months [13.4 – 63.8] |  |
| **BLR_max_** | <median | 4.8 months [3.7 – 7.6] | 0.42 | 35.1 months [22.5 – 54.2] | 0.047* |
|  | ≥median | 3.0 months [2.5 – 6.4] |  | 11.6 months [9.5 – 26.1] |  |

* Statistically significant difference

SLR, spleen-to-liver SUV ratio; BLR, bone marrow-to-liver SUV ratio; PFS, progression-free survival; OS, overall survival.

**Supplementary Figure 1** Kaplan-Meier estimates of PFS according to colonic and splenic SUV_mean_ (A) and SUV_max_ (B) as well as estimates of OS according to colonic and splenic SUV_mean_ (C) and SUV_max_ (D). The numbers of patients at risk in each group and for the respective time-points are shown below the plots.





**Supplementary Figure 2** Kaplan-Meier estimates of PFS according to colonic and bone marrow SUV_mean_ (A) and SUV_max_ (B) as well as estimates of OS according to colonic and bone marrow SUV_mean_ (C) and SUV_max_ (D). The numbers of patients at risk in each group and for the respective time-points are shown below the plots.
